# 840 Limb Salvage After Lomentospora Osteomyelitis in a Burn-Injured Patient Treated with Fosmanogepix

**DOI:** 10.1093/jbcr/iraf019.371

**Published:** 2025-04-01

**Authors:** Gelina Sani, Ian Powelson, Derek Bays, Sierra Young, Erin Louie, Jason Heard

**Affiliations:** University of California Davis; University of California Davis; University of California Davis; University of California Davis; University of California Davis; University of California Davis

## Abstract

**Introduction:**

Burn patients are highly susceptible to infections due to factors like loss of the protective skin barrier, immunosuppression, prolonged open wounds, mechanical ventilation and invasive lines. Fungal infections are a significant cause of late-onset morbidity and mortality. Lomentospora prolificans is a virulent fungus with intrinsic resistance to many antifungal agents. Fosmanogepix, a novel antifungal prodrug of manogepix, targets glycosylphosphatidylinositol (GPI)-anchored proteins essential for fungal cell survival. It has demonstrated efficacy against fungi resistant to other antifungal agents, such as Lomentospora prolificans. This case highlights the use of fosmanogepix for a resistant Lomentospora prolificans infection in a burn patient.

**Methods:**

A 47-year-old male with 40% total body surface area third- and fourth-degree burns was admitted to the burn unit. He required initial escharotomies and fasciotomies followed by serial wound excision and debridement operations including a right lower extremity above the knee amputation. His course was complicated by multiple episodes of acute respiratory distress syndrome, septic shock and progressive tissue ischemia. On hospital day 88, there was clinical suspicion for mold for which tissue cultures and bone biopsies were obtained from his left lower extremity which identified Lomentospora. Combination therapy of voriconazole and terbinafine was initiated, followed by compassionate use of fosmanogepix.

**Results:**

The Lomentospora prolificans culture was resistant to common antifungals such as amphotericin B, itraconazole, and voriconazole. Given his above the knee amputation on the contralateral extremity, limb salvage was endeavored with combination therapy of voriconazole and terbinafine as well as serial excisions to stabilize the wound until fosmanogepix was procured. Fosmanogepix was initiated on hospital day 124, and no mold growth was observed in subsequent cultures. He was ultimately able to be skin grafted and did not require a left lower extremity amputation. The patient was transitioned to oral fosmanogepix and discharged to a rehabilitation facility on hospital day 158.

**Conclusions:**

Fosmanogepix, an investigational antifungal, was successfully used to treat Lomentospora prolificans infection in a burn patient resistant to standard antifungal therapies. This case demonstrates the potential of fosmanogepix as an alternative therapy for resistant fungal infections, offering hope for future management of similar cases.

**Applicability of Research to Practice:**

Burn patients are at increased risk for fungal infections. When infections by resistant organisms such as Lomentospora prolificans occur, early recognition and timely use of novel antifungals like fosmanogepix may be critical for improving outcomes. This case underscores the importance of considering investigational therapies in the management of difficult-to-treat infections.

**Funding for the Study:**

N/A

